# Combined Analysis of CSF Tau, A*β*42, A*β*1–42% and A*β*1–40^ox^% in Alzheimer's Disease, Dementia with Lewy Bodies and Parkinson's Disease Dementia

**DOI:** 10.4061/2010/761571

**Published:** 2010-08-24

**Authors:** Mirko Bibl, Hermann Esselmann, Piotr Lewczuk, Claudia Trenkwalder, Markus Otto, Johannes Kornhuber, Jens Wiltfang, Brit Mollenhauer

**Affiliations:** ^1^Department of Psychiatry, Psychotherapy and Addiction Medicine, Kliniken Essen-Mitte, University of Duisburg-Essen, Henricistrasse 92, 45136 Essen, Germany; ^2^Department of Psychiatry, Psychotherapy, Rheinische Kliniken Essen, University of Duisburg-Essen, 45147 Essen, Germany; ^3^Department of Psychiatry and Psychotherapy, University of Erlangen, Schwabachanlage 6, 91054 Erlangen, Germany; ^4^Paracelsus-Elena Klinik, University of Goettingen, 34128 Kassel, Germany; ^5^Institute for Neurology, University of Ulm, 89075 Ulm, Germany

## Abstract

We studied the diagnostic value of CSF A*β*42/tau versus low A*β*1–42% and high A*β*1–40^ox^% levels for differential diagnosis of Alzheimer's disease (AD) and dementia with Lewy bodies (DLB), respectively. CSF of 45 patients with AD, 15 with DLB, 21 with Parkinson's disease dementia (PDD), and 40 nondemented disease controls (NDC) was analyzed by A*β*-SDS-PAGE/immunoblot and ELISAs (A*β*42 and tau). A*β*42/tau lacked specificity in discriminating AD from DLB and PDD. Best discriminating biomarkers were A*β*1–42% and A*β*1–40^ox^% for AD and DLB, respectively. AD and DLB could be differentiated by both A*β*1–42% and A*β*1–40^ox^% with an accuracy of 80% at minimum. Thus, we consider A*β*1–42% and A*β*1–40^ox^% to be useful biomarkers for AD and DLB, respectively. We propose further studies on the integration of A*β*1–42% and A*β*1–40^ox^% into conventional assay formats. Moreover, future studies should investigate the combination of A*β*1–40^ox^% and CSF alpha-synuclein for the diagnosis of DLB.

## 1. Introduction

Reduced amyloid-*β* (A*β*) 42 peptide concentrations and elevated tau levels in cerebrospinal fluid (CSF) represent supportive features of Alzheimer's dementia (AD) diagnosis [1]. These biomarkers have shown their major diagnostic value in comparison of AD to controls, but overlapping values have hampered sufficient diagnostic accuracy in differentiating other kinds of dementia, especially vascular dementias and dementia with Lewy bodies [2]. The specificity of A*β*42 in the differential diagnosis of AD and other dementias could be improved by measuring the relative A*β*1–42 concentration in CSF as compared to the sum of the peptides A*β*1–40, A*β*1–38, A*β*1–37, A*β*1–39, and oxidized A*β*1–40 (A*β*1–40^ox^) as a percentage value (A*β*1–42%) [3]. Moreover, the percentage value of A*β*1–40^ox^ (A*β*1–40^ox^%) has been proposed as a potentially helpful CSF biomarker in diagnosing DLB [3, 4]. 

This study investigates the additional diagnostic value of these novel CSF biomarker candidates as compared to the well-acknowledged combined analysis of tau and A*β*42 in differentiating the dementias AD, DLB, and PDD. For this purpose, CSF levels of tau, A*β*42, A*β*1–42%, and A*β*1–40^ox^% were determined in CSF of 45 patients with probable AD, 15 with probable DLB, 21 with PDD and 40 nondemented disease controls (NDC). Their respective diagnostic accuracies for each relevant differential diagnostic quest were analyzed. 

## 2. Patients and Methods

### 2.1. Patients

We investigated 121 CSF samples that were referred to our laboratory between 1999 and 2004. CSF concentrations of tau, A*β*42, A*β*1–42%, and A*β*1–40 were measured. Aliquots of these samples had been studied previously under another objective and focussing a distinct issue of differentially diagnosing dementias [3].

CSF of patients with DLB and PDD, respectively, came from the Paracelsus-Elena Klinik, Kassel, a hospital specialized in the management of movement disorders. CSF samples of AD patients and nondemented disease controls came from the memory clinic and from wards at Goettingen University.

A psychiatrist and a neurologist rendered diagnoses based on thorough clinical examination, neuropsychological assessment, clinical records, and best clinical judgment. The investigators were blinded to the neurochemical outcome measures. Investigations were carried out with the informed consent of patients or their authorized caregiver. The study was conducted under the guidelines of the Declaration of Helsinki [5] and approved by the ethics committee of the University of Goettingen and Hessen.

The nondemented disease controls consisted of two subgroups.

#### 2.1.1. Neurological Diseases without Dementia Syndrome

The 15 patients (6 women and 9 men) underwent lumbar puncture for routine investigation of central nervous affection. The patients were suffering from Parkinson's disease (*n* = 6), polyneuropathy (*n* = 2), genetically reconfirmed Huntington's disease (*n* = 2), spinocerebellar ataxia (*n* = 2), peripheral facial nerve palsy (*n* = 1), autosomal dominant hereditary spastic spinal palsy (*n* = 1) and amyotrophic lateral sclerosis (*n* = 1). The Mini Mental Status Examination (MMSE) score in patients with cognitive complaints (*n* = 8) was 28.0 ± 1.5 (mean ± SD). None of these patients displayed clinical features of dementia syndrome DSM IV or NINCDS-ADRDA criteria [6]. Age of this subgroup was 66.7 ± 6.9 years (mean ± SD).

#### 2.1.2. Depressive Cognitive Complainers

The 25 depressive patients (16 women and 9 men) underwent lumbar puncture for differential diagnosis of cognitive complaints during the course of disease. The diagnosis of depression was made according to the criteria of DSM IV and cognitive impairment was assessed by MMSE at minimum. Patients with persistent cognitive decline for more than six months, MMSE score below 26 or clear focal atrophy in brain imaging (CT or MRI) were excluded. Age of this subgroup was 63.2 ± 10.4 years (mean ± SD).

#### 2.1.3. Patients with Alzheimer's Disease

45 patients (27 women and 18 men) fulfilled DSM IV criteria and NINCDS-ADRDA criteria for clinical diagnosis of AD [6]. Structural (CT or MRI) or functional (SPECT or PET) brain imaging displayed global cortical atrophy, or temporal, parietotemporal, or frontotemporal focal atrophy, or marked hypometabolism of these regions.

#### 2.1.4. Patients with Dementia with Lewy Bodies (DLB) and Parkinson's Disease Dementia (PDD)

Dementia with Lewy bodies (DLB, *n* = 15, 3 women and 12 men) was diagnosed according to the consensus criteria [7]. Patients presented with at least two core features according to the criteria and with parkinsonism less than one year before onset of dementia. Enrolled patients were hospitalized for several days to evaluate fluctuating cognition, extrapyramidal symptoms, and visual hallucinations.

Parkinson's disease dementia (PDD) was diagnosed in 21 patients (6 women and 15 men) according to UK Parkinson's Disease Society Brain Bank clinical diagnostic criteria for idiopathic Parkinson's disease and the consensus criteria [7, 8]. All patients presented parkinsonism at least one year before onset of dementia.

The mean age and MMSE score of patient groups are given in Table 1.

### 2.2. Test Methods

#### 2.2.1. Preanalytical Treatment of CSF

CSF was drawn by lumbar puncture into polypropylene vials and centrifuged (1000 g, 10 min, 4°C). Aliquots of 200 *μ*L were kept at room temperature for a maximum of 24 hours before storage at −80°C for subsequent A*β*-SDS-PAGE/immunoblot. The samples were not thawed until analysis. The freezers had an automatic temperature control and alarm system, so that relevant temperature changes during the time of storage can be excluded. CSF for total A*β* and tau ELISA analysis was stored at +4°C and analyzed within two days. The protocol of preanalytical CSF handling was harmonized between the two centres of Goettingen and Kassel.

#### 2.2.2. ELISA for Total-Tau and A*β* 1–42

The ELISAs Innotest hTAU Antigen ELISA and Innotest *β*-Amyloid_(1−42),_ ELISA Innogenetics (Ghent, Belgium) served for quantification of CSF tau and A*β* 1–42, respectively. Both ELISAs were conducted according to published standard methods [9].

### 2.3. A*β*-SDS-PAGE/Immunoblot

A*β* peptide patterns were analyzed by A*β*-SDS-PAGE/immunoblot. For separation of A*β* peptides and subsequent detection, 10 *μ*l of unconcentrated CSF were boiled in a sample buffer for SDS-PAGE, and A*β*-SDS-PAGE/immunoblot was conducted as published elsewhere [10, 11]. CSF samples of each individual patient were run as triplicates. Bands were quantified from individual blots of each patient relative to a four point dilution series of synthetic A*β* peptides using a charge coupled device camera. The detection sensitivity was 0.6 pg (A*β*1–38, A*β*1–40) and 1 pg (A*β*1–37, A*β*1–39, A*β*1–42), respectively. Signal acquisition was linear within a range of 3.8 magnitudes of order [10]. The inter- and intra-assay coefficients of variation for 20 to 80 pg of synthetic A*β* peptides were below 10% [10, 11].

### 2.4. Statistical Analysis

A*β* peptide and tau levels were expressed as absolute values (ng/ml). The data on A*β* peptide levels were obtained from individual blots of each patient. A*β* peptide values were determined in absolute (ng/ml) and percentage values relative to the sum of all investigated A*β* peptides (A*β*1–*X*%). We have characterized patient groups by mean and standard deviation (SD). 

The Mann-Whitney *U*-test was employed for comparisons of diagnostic groups. Multiple comparisons were not performed. Correlations of measured values were estimated by Spearman's Rho. The two-sided level of significance was taken as *P* < .05. The global diagnostic accuracies were assessed by the area under the curve (AUC) of receiver operating characteristic curve (ROC). Cutoff points were determined at the maximum Youden index [12], providing a sensitivity of ≥80%. The statistical software package SPSS, version 12.0, was used for computations.

## 3. Results

### 3.1. Test Results

The mean age of NDC was significantly younger than each of the dementia groups (*P* < 5 × 10^−2^). The dementia groups did not significantly differ from each other in age. The mean MMSE score did not significantly differ between the dementia groups.

Patients with neurological diseases without dementia syndrome exhibited higher levels of CSF A*β*1–40^ox^%  (*P* = 6.1 × 10^−4^) and lower levels of A*β*1–42 (*P* = 1.3 × 10^−2^) than depressive cognitive complainers. Nevertheless, for simplification, statistical analysis considered the two groups as one (NDC).

Table 1 summarizes mean age, MMSE, as well as CSF total tau, A*β*42, A*β*1–42%, and A*β*1–40^ox^% levels of the diagnostic groups.

#### 3.1.1. Neurochemical Phenotype of AD versus NDC

AD was characterized by decreased values of A*β*42 (*P* = 1.8 × 10^−10^) and A*β*1–42%  (*P* = 2.8 × 10^−15^). In contrast, tau (*P* = 4.8 × 10^−10^) and A*β*1–40^ox^%  (*P* = 1.1 × 10^−2^) were elevated in AD.

#### 3.1.2. Neurochemical Phenotype of DLB versus NDC

DLB patients showed lower levels of A*β*42 (*P* = 3.3 × 10^−6^) and A*β*1–42%  (*P* = 2.3 × 10^−5^), but higher A*β*1–40^ox^% concentrations than NDC (*P* = 9.0 × 10^−6^). Tau levels tended to be increased, but failed the level of significance.

#### 3.1.3. Neurochemical Phenotype of PDD versus NDC

PDD patients showed lower levels of A*β*42 (*P* = 1.4 × 10^−4^) and A*β*1–42% (P = 1 × 10^−5^) than NDC. A*β*1–40^ox^% was elevated in PDD (P = 1.7 × 10^−2^). Tau was unchanged between PDD and NDC.

#### 3.1.4. Neurochemical Phenotype of AD versus DLB and PDD

AD displayed lower A*β*1–42% levels than DLB (*P* = 5.9 × 10^−7^) and PDD (*P* = 4.2 × 10^−7^). A*β*42 levels did not significantly differ from DLB and PDD. A*β*1–40^ox^% was lowered in DLB (*P* = 2.6 × 10^−6^), but did not significantly differ from PDD. Tau levels were elevated in AD as compared to DLB (*P* = 2.8 × 10^−3^) and PDD (*P* = 7.1 × 10^−5^), respectively.

#### 3.1.5. Neurochemical Phenotype of DLB versus PDD

The main differences were elevated levels of A*β*1–40^ox^% in DLB (*P* = 1.3 × 10^−3^). A*β*42 was lower in DLB (*P* = 3.0 × 10^−2^), whereas A*β*1–42% and tau were not significantly altered among the two groups.

### 3.2. Correlations

Analysis of each diagnostic group gave the following significant correlations. In NDC, A*β*42 and A*β*1–42% were positively correlated to each other. Higher values of A*β*1–40^ox^% were correlated with male sex. Negative correlations were observed between A*β*1–42% and age as well as A*β*1–40^ox^% and MMSE score. In AD, A*β*42 was positively correlated with A*β*1–42% and male sex, respectively. In PDD, A*β*42 was positively correlated with A*β*1–42%, but negatively with tau levels. No significant correlations were observed in the DLB group. 

### 3.3. Estimates

**Figure 1:**
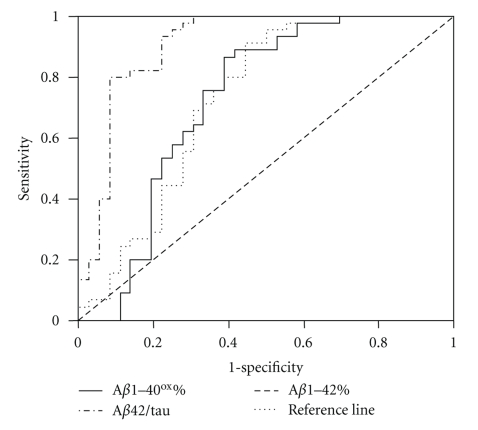
Receiver operator curves for detection of AD among DLB and PDD as a combined group using A*β*42/tau, A*β*1–42% and A*β*1–40^ox^%, respectively.

**Figure 2:**
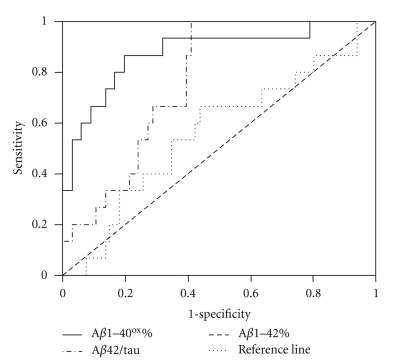
Receiver operator curves for detection of DLB among AD and PDD as a combined group using A*β*42/tau, A*β*1–42% and A*β*1–40^ox^%, respectively.

**Figure 3:**
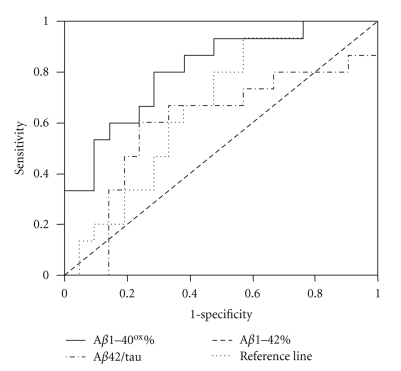
Receiver operator curves for differentiating DLB from PDD using A*β*42/tau, A*β*1–42% and A*β*1–40^ox^%, respectively.

The results of ROC analysis for each relevant differential diagnostic testing are summarized in Table 2. Figures 1–3 show Receiver operator curves for the most relevant differential diagnostic testings.

## 4. Discussion

### 4.1. Biomarker Patterns in the Different Dementia Groups

In agreement with numerous previous studies, we found high levels of tau accompanied by low CSF A*β*42 levels in AD in contrast to nondemented disease controls [2, 13]. In DLB and PDD, these biomarkers displayed a rather unspecific pattern: tau proteins have been found to be in a normal range or slightly increased, paralleled by mildly to moderately decreased CSF A*β*1–42 levels [14–19]. Rises of CSF tau levels have also been detected in Creutzfeldt-Jakob Disease (CJD), vascular dementias and after acute stroke [13, 20, 21], indicating tau to be a sensitive biomarker for neurodestruction, but unspecific for the underlying disease process. The range of results for tau levels in DLB and PDD may result from some unexpected variance of values depending on the actual dynamic of neuronal decay at the time of lumbar puncture. Moreover, clinical diagnosis of DLB and PDD may be confounded with AD and vice versa. The selection of control groups varies among the different studies. In the present study, we compare dementia groups to diseased controls that include neurodegenerative disorders, like Parkinson's disease. This may lead to a higher overlap of CSF tau values than in studies in which healthy controls served for comparison. Especially, when taking into account that PDD may be considered as a clinical state of Parkinson's disease.

The decrease of raw CSF A*β*42 concentrations can also be found in dementias other than AD, but then often in the wake of an overall drop of CSF A*β* peptides [3, 4]. In contrast, the selective decrease of the A*β*1–42 concentration as compared to constant A*β*-overall concentrations is more specific for AD [3]. In line with previous results, the diagnostic accuracy between AD and other dementias could be clearly improved by scaling A*β*42 as a percentage portion of the sum of all investigated A*β* peptides (A*β*1–42%) [3]. 

Regarding A*β*1–40^ox^%, the present study confirms our previous results of its elevated CSF levels in DLB [4]. Remarkably, A*β*1–40^ox^% was only mildly elevated in AD and PDD as compared to controls, leading to a considerably smaller area of overlapping values in comparison to DLB. 

### 4.2. Diagnostic Accuracies for AD and DLB Using A*β*42/Tau, A*β*1–42% and, A*β*1–40^*ox*^%, Respectively

According to the references of The Working Group on “Molecular and Biochemical Markers of Alzheimer's Disease” [22], reasonable diagnostic accuracies of the tau/A*β*1–42 ratio have been reported for detecting AD among nondemented, either healthy or diseased controls [23]. The specificity of this marker combination declined considerably down to 58% when differentiating AD from other neurodegenerative dementias, due to a large overlap of values [9]. We found similar results in the present study. In contrast, disease specific changes of CSF A*β* peptide patterns in AD and DLB enabled higher accuracies for their differential diagnosis, also in discrimination to PDD. With accuracies of 80% at minimum, low CSF levels of A*β*1–42% were the most accurate biomarker for diagnosing AD among PDD alone and in a combined group of DLB and PDD. For the differentiation of AD from DLB, A*β*1–42% and A*β*1–40^ox^% yielded comparable accuracies of 80% at minimum. The differential diagnosis of DLB and PDD could be made at a sensitivity and specificity of 80% and 71%, respectively, using A*β*1–40^ox^% as the most accurate biomarker. These accuracies fall within the range of the aforementioned requirements or come close to it [22]. 

The reason for relative A*β* peptide values being superior to raw A*β* levels include: (i) A*β*1–42, but not A*β*1–40/A*β*1–42 showed a *U*-shaped natural course in normal aging [24]; (ii) in contrast to absolute A*β* peptide values, the relative abundances remained largely stable after different preanalytical procedures [11, 25]; (iii) referencing A*β*1–42 to A*β*1–40 avoids false positive and negative AD diagnosis in patients with constitutionally low and high CSF A*β*42 levels, respectively [26]; and (iv) dementias with low A*β*42 levels in the course of an overall decrease of CSF A*β* peptides will be sorted out from the diagnosis of AD [3]. The whole amount of CSF A*β* peptides measurable in the A*β*-SDS-PAGE/immunoblot is closely correlated to CSF A*β*1–40 levels [26]. This makes it possible to insert the ratio A*β*1–42/A*β*1–40 as a substitute for A*β*1–42%. Thus, the above considerations apply to both A*β* peptide ratios and percentage A*β* peptide values.

### 4.3. Conclusions

We consider CSF A*β*42/tau to be a sensitive biomarker for detection of AD, but not specific enough for excluding other forms of dementia, like DLB and PDD. Yielding contrasts of 80% or greater, decreased CSF A*β*1–42% and elevated A*β*1–40^ox^% are promising biomarker candidates for AD and DLB, respectively. However, the pathophysiological meaning of these biomarkers in the development of AD and DLB remains to be clarified.

The further progress of A*β*-peptide patterns as applicable biomarkers requires validation in independent studies on neuropathologically confirmed cases. Under this respect, we recently showed that A*β*1–40^ox^% does not differ among clinically and neuropathologically defined cases of DLB [27]. The major component of Lewy bodies, *α*-synuclein, displayed reduced CSF levels in Parkinson's disease and DLB as compared to AD and controls [28, 29]. For future studies on differentially diagnosing DLB, we propose the investigation of combined CSF *α*-synuclein and A*β*1–40^ox^% levels. Furthermore, there is a need for translating the measurement of A*β*1–42% and A*β*1–40^ox^% into more common assay formats, like ELISA [30]. 

### 4.4. Limitations of the Study

Our results are limited by the reliance on clinical diagnosis results, because of potential misclassification. Another point of concern is the size of patient groups for DLB and PDD.

## Figures and Tables

**Table 1:** Age, MMSE, Total tau, A*β*42, A*β*1–42%, and A*β*1–40^*ox*^% in the CSF of the diagnostic groups.

Diagnosis	NDC (*n* = 40)	AD (*n* = 45)	DLB (*n* = 15)	PDD (*n* = 21)
	mean ± SD	mean ± SD	mean ± SD	mean ± SD
Age	64.5 ± 9.3	70.9 ± 9.2	71.4 ± 7.6	73.2 ± 7.2
MMSE	28.6 ± 1.4	19.4 ± 5.8	19.2 ± 3.0	18.1 ± 7.2
Total tau (ELISA)^1^	0.23 ± 0.14	0.62 ± 0.34	0.37 ± 0.29	0.31 ± 0.24
A*β*1–42 (ELISA)^1^	0.79 ± 0.27	0.41 ± 0.14	0.37 ± 0.17	0.51 ± 0.22
A*β*42/Tau (ELISA)^1^	4.74 ± 3.03	0.87 ± 0.58	1.63 ± 1.35	3.16 ± 2.72
A*β*1–42% (A*β*-SDS-PAGE/immunoblot)^2^	11.65 ± 3.53	4.38 ± 0.89	7.13 ± 2.13	7.54 ± 2.07
A*β*1–42% (A*β*-SDS-PAGE/immunoblot)^2^	0.77 ± 0.5	0.88 ± 0.27	1.78 ± 0.70	1.05 ± 0.48

^1^A*β* peptide concentrations as measured by ELISA (ng/ml or ratio)

^2^A*β* peptide values of A*β*1–42 and A*β*1–40^ox^, respectively, relative to the sum of all measurable A*β* peptides in the A*β*-SDS-PAGE/ immunoblot

**Table 2:** Cutoff points, sensitivities, and specificities.

differential diagnosis	Parameter	cut off	sensitivity	specificity	Youden index	AUC	95%-CI
AD versus DLB	A*β*42/tau	1.163	80%	53%	0.33	0.664	0.483–0.845
	A*β*1–42%	5.093	80%	100%	0.80	0.933	0.872–0.994
	A*β*1–40^ox^%	1.144	89%	87%	0.76	0.908	0.802–1.014

AD versus PDD	A*β*42/tau	1.450	91%	67%	0.58	0.775	0.630–0.919
	A*β*1–42%	5.730	93%	86%	0.79	0.889	0.773–1.005
	A*β*1–40^ox^%	1.104	87%	43%	0.30	0.592	0.420–0.763

AD versus DLB and PDD	A*β*42/tau	1.450	91%	56%	0.47	0.728	0.610–0.747
	A*β*1–42%	5.093	80%	92%	0.72	0.907	0.834–0.981
	A*β*1–40^ox^%	1.104	87%	61%	0.48	0.723	0.600–0.847

DLB versus PDD	A*β*42/tau	3.229	93%	43%	0.36	0.663	0.487–0.840
	A*β*1–42%	8.855	80%	33%	0.13	0.597	0.395–0.799
	A*β*1–40^ox^%	1.244	80%	71%	0.51	0.810	0.667–0.952

DLB versus AD and PDD	A*β*42/tau	0.546	80%	26%	0.06	0.560	0.396–0.723
	A*β*1–42%	5.198	87%	61%	0.59	0.765	0.658–0.871
	A*β*1–40^ox^%	1.144	87%	80%	0.67	0.877	0.769–0.985

## Abbreviations

A*β* peptides: amyloid-beta peptides
A*β*-SDS-PAGE/immunoblot: amyloid-beta-sodium-dodecyl-sulphate-polyacrylamide-gel electrophoresis with western immunoblot
AD: Alzheimer's disease
CCD-camera: charge coupled device camera
CSF: cerebrospinal fluid
DLB: dementia with Lewy bodies
ECL: enhanced chemiluminescence
ELISA: Enzyme Linked Immunosorbent Assay
MMSE: Mini-Mental-Status Examination
NINCDS-ADRDA: National Institute of Neurological and Communicative Disorders and Stroke-Alzheimer's Disease and Related Disorders Association
NDC: nondemented disease controls
PDD: Parkinson's disease dementia
SDS: sodium dodecyl sulphate.
